# *Streptococcus pyogenes* (“Group A Streptococcus”), a Highly Adapted Human Pathogen—Potential Implications of Its Virulence Regulation for Epidemiology and Disease Management

**DOI:** 10.3390/pathogens10060776

**Published:** 2021-06-21

**Authors:** Nikolai Siemens, Rudolf Lütticken

**Affiliations:** 1 Department of Molecular Genetics and Infection Biology, Interfaculty Institute for Genetics and Functional Genomics, University of Greifswald, 17487 Greifswald, Germany; nikolai.siemens@uni-greifswald.de; 2 DWI Leibnitz-Institute for Interactive Materials, 52074 Aachen, Germany

**Keywords:** *Streptococcus pyogenes*, group A streptococcus, virulence regulation, transcriptional factors, two components systems

## Abstract

*Streptococcus pyogenes* (group A streptococci; GAS) is an exclusively human pathogen. It causes a variety of suppurative and non-suppurative diseases in people of all ages worldwide. Not all can be successfully treated with antibiotics. A licensed vaccine, in spite of its global importance, is not yet available. GAS express an arsenal of virulence factors responsible for pathological immune reactions. The transcription of all these virulence factors is under the control of three types of virulence-related regulators: (i) two-component systems (TCS), (ii) stand-alone regulators, and (iii) non-coding RNAs. This review summarizes major TCS and stand-alone transcriptional regulatory systems, which are directly associated with virulence control. It is suggested that this treasure of knowledge on the genetics of virulence regulation should be better harnessed for new therapies and prevention methods for GAS infections, thereby changing its global epidemiology for the better.

## 1. Introduction

Among the Gram-positive cocci, *Streptococcus pyogenes* (colloquially termed the “group A streptococcus” or “GAS”, based on the presence the group A cell wall polysaccharide antigen), is one of the most successful pathogens worldwide. It causes superficial and deep (invasive) infections almost exclusively in humans. Among those are the quite common upper respiratory tract infections (pharyngotonsillitis—“sore throat”) predominantly occurring in children [1], superficial skin infections (*impetigo*), and deep skin infections, such as erysipelas and cellulitis [2], and invasive infections appearing as necrotizing fasciitis (NF, “flesh-eating disease”, necrotizing soft tissue infections—NSTI [3]) or sepsis. In addition, superficial and invasive GAS infections can be associated with reactions to toxins produced by *S. pyogenes,* appearing (among other signs) as scarlet fever (*scarlatina*) [1] or as streptococcal toxic shock syndrome (STSS) [4]. Moreover, late non-suppurative sequelae might appear weeks after a streptococcal infection, namely the acute rheumatic fever (ARF, including rheumatic heart disease—RHD [5]) primarily after throat infections or an acute glomerulonephritis (AGN, affecting the kidney) occurring after both streptococcal throat and skin infections [6]. These sequelae are ascribed to autoimmune reactions directed against cross-reactive streptococcal antigens or neoantigens developing during an acute GAS infection [7].

## 2. Epidemiology, Clinics, Therapy, and Prevention

Since GAS infections are found worldwide in humans, they are considered a global health problem [8]. However, the distribution among different populations is determined by differences in the GAS strain virulence factor armory, in a very prominent manner by the M-protein [9], and the social environment of the affected population [10]. Therefore, the determinants for the occurrence, the prevalence, and the severity of the diverse non-invasive and invasive GAS diseases are to be searched for in the microorganism, in the host, and in the environment, including the socioeconomic conditions of the affected population.

On the one hand, the diversity of the GAS major surface protein M (determined by the *emm* gene), with more than 230 types [11], and eliciting protective antibodies in the affected patient—early recognized by Rebecca Lancefield [12]—assures its rapid distribution among susceptible populations. The main routes of transmission are: (I) droplet infection, predominantly for upper respiratory tract (URT) disease, but also for superficial and deep skin and wound infections, and (II) direct contact for both respiratory and invasive infections [13]. For an infection with GAS to be successfully established, the presence of a certain M-protein and other associated virulence traits, the individual’s acquired immunity, directed against the respective M-protein or other bacterial constituents, and host genetics, are of importance. Of course, the kind of lesion (superficial or deep) also determines if the infection remains restricted to the throat or superficial skin or results in a severe invasive infection.

For the non-suppurative sequelae (ARF, RHD, AGN) early data suggest, that “rheumatic fever M-types” exist, which may cause the majority of cases. Based on recent data, a greater diversity of M-types (if not all) can cause ARF and RHD [14,15]. For the clinical establishment of ARF and RHD, it is known by former family and genetic studies that the individual host susceptibility plays a crucial role [16]. This concept is supported by recent data from the South Pacific area and from Australia [17,18]. However, we have still to learn what determines the individual symptoms in the post-streptococcal autoimmune diseases, e.g., if they are accompanied by certain symptoms, such as Sydenham’s chorea or a PANDAS (pediatric autoimmune neuropsychiatric disorder associated with streptococcal infections) [19,20].

We also must take into account other conditions, which might contribute to the epidemiology of GAS infections, namely the fact that GAS carriers exist that may act as sources. The GAS carrier state can still be called an enigma [21], although some molecular data suggest what may contribute to its occurrence [22,23].

The carrier state may be interconnected with treatment failures that do occur in spite of the—still rather universal—sensitivity of GAS strains for penicillin and other suitable beta-lactams [24]. Different phenomena are discussed as causing treatment failures: resistances to macrolides, which are recommended as alternative treatment of URT infections for children with penicillin hypersensitivity, can lead to failure [25]; beta-lactamases produced by co-infecting bacteria, such as *Staphylococcus aureus*, could inactivate the penicillin given. Other factors, such as biofilm formation [26,27] or intracellular survival [28] of GAS, could also contribute, but not all causes for treatment failures are known yet [29,30]. However, the hitherto generally accepted beta-lactam sensitivity of GAS may not be given forever; first reports on GAS strains with reduced penicillin sensitivity are alarming [31,32].

In our opinion, thus far, no convincing data were presented in the literature in which the effective antibiotic treatment of URT GAS infections, recommended for a long time, could be abstained from without risking the development of serious sequelae-like ARF/RHD. Moreover, invasive infections could suddenly develop from primarily superficial and rather tame infections into often deadly invasive disease. Therefore, in addition to avoiding exposure to an infected person, prevention of GAS infections must be the ultimate goal. Thus far, no licensed GAS vaccine is available. However, considerable progress in the preclinical development of a candidate vaccine has been made in recent years; most of these vaccines are based on peptide sequences derived from M-proteins, but other approaches are also being evaluated (for review, see [33]).

This review focuses on established virulence factors of GAS and their regulation, which play a major role in the different diseases caused by this versatile pathogen. In view of the—partially justified—reserve to treat every URT infection with an antibiotic, and the threat of antibiotic resistances developing even in GAS, deeper knowledge about the determinants of virulence may help to develop new “anti-virulence” agents for successful “antibiotic-free” treatments [34,35], or to be used as adjunct therapeutics in severe invasive GAS infections, as proposed recently for combination therapy of *Staphylococcus aureus* infections [36]. Other non-antibiotic antimicrobial agents directed against streptococcal constituents, which are not (yet) established as virulence regulators, might also be developed. Those are not in the scope of this review. For example, inhibitors of sortase A may indirectly influence virulence by interfering with the attachment mediated by surface proteins and thereby being important for the epidemiology [37]. Inhibitors or inducers of streptococcal quorum sensing systems may also influence the GAS colonization status, either directly or indirectly through action on bacterial competitors in the throat or on the skin [38,39].

## 3. Major GAS Virulence Factors

GAS infections are complex and multifactorial processes, and both host and bacterial factors are crucial for successful establishment of an infection. The ability of GAS to colonize the human host and to establish an initial infection can be primarily attributed to the surface located virulence factors. Secreted factors allow the bacteria to disseminate to the deeper layers of the tissue and help to evade an orchestrated host immune response. In this section, we will summarize major GAS virulence factors involved in these processes.

### 3.1. GAS Adhesins

The adhesion of GAS to different epithelial cells is believed to be a two-step process. First, lipoteichoic acid mediates a weak, reversible, and unspecific interaction with epithelial surfaces [40]. The second step of adhesion involves surface anchored and surface associated proteins. These adhesins either bind directly to the human host cell receptors or use matrix and/or plasma proteins as bridging molecules [41]. Streptococcal M protein is the most abundant surface anchored protein of GAS and probably one of the best-characterized virulence factors. A plethora of proteinaceous and non-proteinaceous interaction partners is described, e.g., M protein binds directly to CD46 on human keratinocytes, it uses fibronectin as a prime target on epithelial cells [42,43], and interacts with glycosaminoglycans on human skin fibroblasts [44]. The interactions of M protein with these three ligands are just a few of many examples of the ability of GAS to adhere to host cells. There are many other classical and non-classical GAS adhesins. These include several fibronectin binding proteins [45], collagen binding protein Cpa [46], vitronectin binding protein [47], and plasminogen binding proteins [48,49,50,51,52]. It should be noted that not all of them are expressed by all GAS serotypes, their expression is growth and infection stage dependent, and some of them show cell type specificity [40].

In contrast to the previous assumption that GAS are extracellular pathogens, now it is appreciated that GAS can invade and persist in human cells. LaPenta and colleagues were the first to show that GAS can efficiently internalize into and persist in non-phagocytic cells at frequencies equal to those of *Listeria* and *Salmonella* [53]. GAS use, e.g., the M protein-fibronectin-α_5_β_1_-integrin axis to induce cytoskeletal rearrangement of the host cell. This results in actin accumulation around the pathogen and subsequent trafficking of GAS inside the cell [45]. Intracellularly, GAS can persist in a safe caveosomal compartment or reside inside the phagolysosome [45,54].

### 3.2. GAS Secreted Factors

To disseminate through the tissue and to evade and modulate host immune response, GAS secrete a number of pore-forming toxins, proteases, DNases, and superantigens (SAgs). Several of them are highly upregulated at different stages of infection, suggesting that each of them plays a crucial role in the progress of infection [55,56,57].

Streptolysins S and O (SLS; SLO) are toxins that cause cytolysis through pore-formation. In addition, immunomodulatory functions for these two toxins are described. SLO is an oxygen sensitive, immunogenic cytolysin, which interacts with cholesterol-rich eukaryotic membranes [58]. Recently it was shown that SLO also binds to multiple types of glycans [59]. Oligomerization of SLO on eukaryotic membranes disrupts cytoplasmic membrane integrity of a diverse range of human cells and induces cell death through different mechanisms, including apoptosis, necrosis, and pyroptosis [58,60,61]. In contrast to SLO, SLS is a small non-immunogenic, oxygen stable peptide, which requires a carrier molecule for its activity [62]. It accumulates on membranes of erythrocytes, leukocytes, and platelets, and causes cytolysis by a yet not fully characterized mechanism [63]. It was suggested that SLS interacts with the major erythrocyte anion exchange protein band 3. This interaction induces osmotic change, Cl^-^ influx, and lysis of erythrocytes [64]. Despite their cytolytic activities, immunomodulatory functions are also described for these two toxins. SLS activates sensory neurons to produce pain. This results in a release of neuropeptides, which in turn suppress neutrophil recruitment to the site of infection and subsequent killing of the pathogen [65]. SLO impairs critical neutrophil functions, including phagocytic clearance, degranulation, and formation of neutrophil extracellular traps (NETs) [66].

In addition to the toxins with cytolytic properties, GAS secrete a broad range of proteases. These include immunoglobulin degrading enzymes IdesS and EndoS, streptococcal pyrogenic exotoxin B (SpeB), a non-enzymatic plasminogen activator streptokinase (Ska), and the subtilisin-like proteases SpyCEP and C5a peptidase (ScpA) [67].

IdeS hydrolyzes four subclasses of IgG, whereas EndoS specifically alters IgG Fc glycosylation [68,69]. Subsequently, altered IgG cannot opsonize the pathogen resulting in impaired phagocytosis and pathogen clearance by phagocytic cells. ScpA cleaves human complement components C5a, C3, and C3a resulting in impaired neutrophil activation [70,71]. SpyCEP specifically cleaves CXC chemokines, including CXCL1-3, CXCL5-6, and CXCL8. All these chemokines play a critical role in chemoattraction of eosinophils, neutrophils, and monocytic cells [72,73]. Ska non-enzymatically converts plasminogen to proteolytically active plasmin and, thereby, interferes with host fibrinolytic system allowing the bacteria to spread [74].

In contrast to the proteases mentioned above, SpeB shows a much broader substrate spectrum [67]. On the bacterial side, SpeB removes anchored proteins from the bacterial surface, including the M protein, C5a peptidase, and a range of fibronectin binding proteins [75,76,77]. Furthermore, it hydrolyses secreted virulence factors like EndoS, SLO, and SAgs [78,79,80]. On the host side, SpeB degrades IgA, IgM, IgD, and IgE and cleaves IgG into Fc and Fab fragments [81]. Moreover, SpeB cleaves components of the complement activation pathway [82], IL-1β [83], and degrades a wide range of chemokines, including CXCL1-7, CXCL10-14, CXCL16, CCL20, XCL1, and CX3CL1 [84].

There are many more secreted virulence factors, including DNases, streptococcal inhibitor of complement (SIC), and SAgs [67]. All of them contribute to colonization, immune evasion, immunomodulation, bacterial spread, and other crucial infection relevant functions [85]. To ensure that the right factor is expressed at the precise time point of the infection stage, the expression is tightly controlled by two component systems (TCS) and stand-alone transcriptional regulators.

## 4. Major GAS TCS and Stand-Alone Transcriptional Regulators Involved in Virulence

Thirteen TCS and at least 30 stand-alone transcriptional regulators control gene expression in GAS [86,87,88,89]. They respond to host environmental signals and coordinate an appropriate bacterial response. Nutrient availability, temperature, components of the innate and adaptive immunity, and chemical stressors are a few challenges of the complex and coordinated human host response that the bacteria are facing during the course of infection. In this section, we summarize major TCS and stand-alone regulators, which are directly linked to bacterial infectivity and immune evasion.

### 4.1. GAS TCS Involved in Virulence

Composition is common to almost all TCS. TCS are distributed through the entire chromosome of GAS [90] and consist of two components: a transmembrane sensor histidine kinase and an associated cytoplasmic response regulator. An environmental signaling stimulus is usually sensed by two sensor kinase molecules, which form a dimer. This interaction results in activation and autophosphorylation of histidine residues in the cytoplasmic transducer domain and subsequently, the phosphoryl group is transferred to an aspartyl residue of the associated response regulator. The regulator in turn acts as a transcription controlling factor of gene expression with repressive and/or inducing functions [89].

#### 4.1.1. CovR/S

One of the best-characterized TCS of GAS is control of virulence (CovR/S or capsule synthesis regulator (CsrR/S)). The kinase CovS senses environmental Mg^2+^ and antimicrobial peptide LL-37 and transduces the signals to the transcriptional regulator CovR [91,92]. CovR regulates the expression of several downstream effector regulons involved in carbon utilization, nitrogen metabolism, quorum sensing, biosynthesis, and virulence [89]. In general, CovR/S TCS is considered a master regulator of GAS. Transcription of up to 15% of the entire GAS chromosome is under control of CovR/S [93]. However, it appears that the major control is more of indirect nature. The system acts primarily as a repressor and regulates multiple genes encoding for virulence relevant factors, including SpeB, SLS, SLO, SpyCEP, Ska, EndoS, streptodornase (Sda1), fibronectin binding protein Fba, and hyaluronic acid capsule operon *hasABC* [93,94,95,96,97]. In addition, CovR/S controls up-to 18 transcriptional stand-alone regulators including the RofA-like protein type family (RALP) regulator RivR (Ralp4). RivR controls the expression of capsule relevant genes and G-related α2-macroglobulin-binding protein (Grab) [98,99]. Interestingly, GAS invasiveness is enhanced by spontaneous mutations of the CovR/S TCS [56,100,101,102]. It was shown that mice tissue passage of GAS selects for a 7-bp frame-shift mutation in the *covS* gene [56,101,102]. Subsequently, impaired CovR/S signaling results in upregulation of several virulence factors, including a bacteriophage-encoded DNase Sda1 [102], Ska, SpyCEP [101], and SLO [103]. At the same time, natural *covR/S* mutants lack SpeB expression [56,102]. Loss of SpeB expression results in accumulation of Ska, Sda1, and M protein among others. All these factors contribute to immune evasion and facilitate systemic bacterial spread [79,104,105]. However, direct involvement of SpeB in invasive infections is still under debate. Although some bacteria lose SpeB through *covR/S* mutation, SpeB is still detectable in sera and tissues of NSTI patients [56,106]. Controversial results on the contribution of SpeB to the severity of infection are also reported in mice studies of soft tissue infections. Some reports have shown that SpeB contributes to bacterial dissemination and disease severity [107,108,109,110], while others reported that *speB*-deficient mutant strains are as virulent as their parental wild type strains [111,112].

#### 4.1.2. Ihk/Irr

Several studies indicate that Ihk/Irr TCS of GAS responds to the cellular innate immunity axis [113,114]. Human neutrophils, monocytes, and macrophages play an important role in this axis. They phagocytose bacteria and destroy them through acidification of the phagolysosome, ROS production, granule fusion, or delivery of antimicrobial peptides. Furthermore, neutrophils degranulate or form NETs to kill extracellular bacteria [67]. Voyich and colleagues were the first to show that genes encoding the Ihk/Irr TCS are upregulated if GAS were present inside neutrophils [114]. Subsequently, the authors demonstrated that knockout of *irr* resulted in impaired survival of GAS in neutrophils [114]. These results demonstrated that Ihk/Irr controls expression of genes, which are critical for bacterial survival inside neutrophils. In line with this, Hertzén and colleagues reported that genes encoding the Ihk/Irr TCS are upregulated at early stages of phagocytosis of GAS by human primary macrophages [113]. Over time, *ihk/irr* transcripts diminished and *covR/S* transcripts increased. In addition, the authors showed that *ihk/irr* deficient GAS mutant is more efficiently killed by macrophages as compared to the parental wild type strain [113]. Furthermore, increased transcript levels of both genes, *ihk* and *irr*, were detected in biopsies of NSTI patients [56] and in muscle biopsies of GAS infected non-human primates [115]. Although these four studies indicate that Ihk/Irr TCS is directly linked to GAS pathogenesis and particularly contributes to GAS responses within phagocytes, it is still unknown which intracellular host signals are sensed and which genes are directly controlled by Ihk/Irr TCS.

#### 4.1.3. CiaH/R

Competence induction and altered cefotaxime susceptibility (CiaH/R) TCS is one of the best-characterized TCS in pneumococci. It is involved in competence, protects pneumococcal cell from lysis by antibiotics, regulates the expression of chaperones and heat shock proteins, and regulates the acid tolerance of pneumococci (reviewed in [116]). In contrast, only a limited number of studies investigated the role of this TCS in GAS. A recent study by Kachroo and colleagues showed that transcripts of the CiaH/R TCS were highly upregulated in muscle biopsies of infected non-human primates, indicating that this system might play a role in severe GAS infections [115]. The authors also showed that *ciaH* isogenic deletion mutant was significantly attenuated in mice infections [115]. However, only two studies investigated potential CiaH/R regulatory axis in GAS. Riani et al. showed that CiaH/R TCS responds to Co^2^+, Cu^2+^, and Zn^2+^ stimulations [117]. Furthermore, Tatsuno and colleagues demonstrated that *ciaH* mutant was sensitive to H_2_O_2_ and showed reduced growth rates in acidic media [118]. Based on these results, it is tempting to assume that CiaH/R TCS might play a role in GAS responsiveness to intracellular environment of professional phagocytes. Nonetheless, studies investigating the CiaH/R TCS in GAS infections remain to be performed.

#### 4.1.4. FasBCA/X

The fibronectin/fibrinogen binding/hemolytic activity/streptokinase (Fas) regulatory system was originally identified in a GAS M49 strain [119]. It is transcribed as a polycistronic message that encodes for two histidine kinases FasB and FasC and a response regulator FasA. In addition, a monocistronic non-coding RNA *fasX* of 300 bp, which is under FasA control, is encoded within the operon [119]. Functional FasBCA operon represses the transcription of adhesins (Fbp54 and Mrp) and favors the expression of genes encoding for Ska and SLS [119]. A similar bacterial phenotype was observed when only *fasX* was knocked out. Detailed studies showed that *fasX* binds to *ska* mRNA and these interaction stabilizes *ska* transcription leading to an increased Ska protein expression [120]. In contrast, *fasX* binding to *cpa* transcripts, a gene encoding for a structural component of the pilus, inhibits the access of ribosomes to *cpa* mRNA, and subsequently suppresses Cpa translation [121]. Such actions by *fasX* are believed to regulate the switch of GAS from colonizing to disseminative phenotype. However, all studies described above are based on in vitro experiments and a final proof in an in vivo infection model remains to be shown.

### 4.2. GAS Stand-Alone Regulators Involved in Virulence

In contrast to TCS, stand-alone regulators coordinate the transcription of virulence factors without inputs from a sensor kinase. Whether they act alone or the potential respective kinase was not yet identified, remains unknown. Some of transcriptional regulators have been extensively studied (e.g., RofA-like protein (RALP) family regulators and Mga) whereas others were not. Most of them contain DNA-binding domains, which interact with promotor regions of the genes they control [90].

#### 4.2.1. Multiple Gene Regulator of Group A Streptococci—Mga

One of the best-characterized regulators in GAS is Mga. Mga was first identified by Spanier and colleagues in 1984 [122]. Deletion of this gene in GAS resulted in loss of M protein expression [122]. Later, Mga was confirmed as a positive regulator of the M protein encoding *emm* gene [123], and the associated gene locus comprising multiple genes encoding for the host cell adhesins M and M-like proteins (*fcrA* and *enn*), fibronectin binding protein (*fba*), and immune evasion proteins C5a peptidase (*scpA*), and the secreted inhibitor of complement (*sic*) [90,124]. In general, Mga regulon is activated by elevated CO_2_ levels, increasing temperature, iron-limiting conditions [125,126,127], and glucose availability [128], which suggests that Mga might be a regulator component of a classical TCS and not a stand-alone regulator. Further evidence is provided by the structure of Mga. Mga has two conserved helix-turn-helix (HTH) motifs near the N-terminus [129], two central phosphotransferase system (PTS) regulatory domains (PRD) [130], and a structural homology to the EIIB domains at the C-terminus [131]. Both HTH domains are required for DNA binding and transcriptional activation [130]. Characterization of purified Mga revealed that it could be phosphorylated at conserved histidines within the PRDs in response to different carbon sources [132]. Although several studies demonstrate that Mga is potentially a response regulator, a cognate sensor kinase has never been identifier to date. Mga is expressed by virtually all clinical GAS isolates and two alleles, *mga-1* and *mga-2*, have been identified [133]. The *mga-1* allele is exclusively found in serum opacity factor (SOF) negative strains, while *mga-2* allele is present in SOF positive strains. It has been suggested that the *mga* variants and the structure of adjacent *mga*-locus are linked to the tissue tropism of GAS strains. *Mga-1* allelic strains are mostly associated with throat infections, while *mga-2* allelic strains with skin and systemic infections [134]. In addition to the genes mentioned above, several other genes relevant for virulence, which are not located within the *mga*-locus, are under the Mga control. These include genes encoding for the fibronectin binding surface proteins SOF and SfbX [135,136] and the collagen-like protein SclA [137,138]. This regulatory network suggests that Mga controls virulence factors, which are associated with initial colonization and/or infection of the human host. In line with this it was shown that *mga* transcript abundance increases during the first 24 h of a skin NSTI model infection and declines while the infection is progressing [56]. Furthermore, several studies have shown that *mga* deficient mutant strains are attenuated in in vivo mice infection models as compared to their parental wild type strains [132,139,140].

#### 4.2.2. RALP Family Stand-Alone Regulators

In total, four RALPs have been identified in GAS: RofA (Ralp1), Nra (Ralp2), Ralp3, and RivR (Ralp4) [90]. They are involved in control of GAS-host cell interactions and are mainly expressed during stationary growth phase [89]. Two of them, RofA and Nra, are located within the FCT (fibronectin binding, collagen binding, T-pilus) region. In general, FCT-region is an 11–16 kb combinatorial region located between two conserved genes *hsp*33 and *spy*0136. In addition to the genes mentioned above, genes encoding for sortases and chaperons are also located within the region. Based on the heterogeneity of gene content, nine subtypes, FCT-1-9 are designated [141]. RofA was identified first [142]. It is a positive transcriptional regulator of the FCT1-2 and 4-9 encoding genes for SfbI, Cpa, and T antigen [143]. Furthermore, it represses directly or indirectly transcription of genes encoding for secreted virulence factors SLS, SpeB, and the SAg SpeA [144].

Nra is an exclusive regulator of FCT-3 type GAS strains, which include M3, M5, M18, and M49 serotypes [145]. All other GAS strains possess RofA instead of Nra in this region [145]. Nra is characterized as a repressor of the genes encoded within the FCT region and the capsule biosynthesis operon *hasABC* [146]. Furthermore, it directly or indirectly inhibits the expression of Mga and its regulon, Ihk/Irr TCS, Ralp3, and Ralp4 [146,147].

Ralp3 is encoded within the ERES (*eno*-*ralp3*-*epf*-*sagA*) pathogenicity island [146]. This region is exclusively present in M1, M4, M12, M28, and M49 GAS serotypes, whereas other serotypes lack *ralp3* and *epf* genes [146]. Ralp3 positively regulates the Mga and Nra regulons [146,148]. Ralp4 (RivR), which was identified based on 32% amino acid identity with Nra, enhances transcription of the Mga regulon [98,99].

All of the RALP family regulators seem to primarily control surface anchored and associated factors, which are mainly involved in initial infection of the host cell structures. Knockout of these stand-alone regulators results in reduced bacterial binding to human matrix and plasma proteins, and diminish attachment to and internalization into human epithelial cells [51,99,145,146,148]. However, only a few studies validated these results in vivo or ex vivo. It was shown that *nra* transcription is upregulated in NSTI tissue biopsies [56]. Subsequent analysis revealed that Nra contributes to biofilm formation of GAS in skin tissues. Knockout of *nra* abolished biofilm formation of GAS in an experimental skin tissue model [56]. Ralp3, even if not present in all streptococcal strains, seems to be essential for survival in human blood [148,149]. Furthermore, it was shown that a *ralp3* mutant strain was less virulent in systemic infection of mice as compared to its parental strain [150].

#### 4.2.3. Rgg/RopB

The global gene regulator of proteinase B (RopB) or Rgg is a growth phase-/quorum sensing-dependent global gene regulator of GAS. RopB mainly controls amino acid and non-glucose carbohydrate metabolism pathways of GAS [151,152,153]. However, it also directly or indirectly regulates the expression of virulence factors such as SLO and NADase (*spn*) [153]. Furthermore, RopB controls *speB* expression in cooperation with an eight amino acid leaderless SpeB-inducing peptide (SIP) [154]. At high bacterial density, the secreted SIP peptide is reimported into the bacterial cytosol, where it directly interacts with RopB. It was shown that the SIP-RopB signaling pathway is active during infection and contributes significantly to GAS pathogenesis [154]. Furthermore, the *ropB* gene was identified as a mutational hotspot in GAS. Strains with the mutated *ropB* allele abrogate SpeB expression and are less virulent in murine models of systemic infection [155] and necrotizing myositis [154,156].

#### 4.2.4. PerR

As previously mentioned, the bacteria are constantly exposed to oxidative stress during an infection. Reactive oxygen species (ROS), including O_2_^−^, H_2_O_2_, and OH^−^, are generated by oxidative burst within phagocytes or directly from atmospheric oxygen. As a result, GAS have evolved different protective responses. The manganese-dependent superoxide dismutase (SodA) converts O_2_^−^ into H_2_O_2_ and O_2_ [157]. Since GAS are catalase negative, alkyl hydroperoxide reductase (AhpC), and glutathione peroxidase (GpoA) neutralize H_2_O_2_ [158,159]. Furthermore, iron-chelating protein Dpr (Dps-like peroxide resistance protein) protects GAS against oxidative DNA damage [160]. The three factors mentioned above are under peroxide stress regulator (PerR) control [158,160,161]. PerR is a homolog of the H_2_O_2_- and metal ion-responsive ferric regulator (Fur) of *Bacillus subtilis* [162]. Deletion of *perR*, *ahpC,* or *sodA* attenuated GAS virulence in intraperitoneal and subcutaneous murine models of infection [158,161]. Furthermore, Gryllos and colleagues have shown that deletion of *perR* in an M3 strain was associated with reduced resistance to phagocytic killing [163]. The increased killing of this *perR* mutant was reversed by inhibition of oxidative burst. Moreover, the *perR* mutant was attenuated in a non-human primate model of pharyngitis [163]. A transcriptional analysis between an M14 GAS strain and its *perR* mutant in absence of a stress stimulus revealed that PerR regulates PmtA (PerR-regulated metal transporter A) [164]. A *PmtA* knockout mutant was more susceptible to killing by H_2_O_2_. However, the *pmtA* gene was not found upregulated in an in vivo mouse model of soft-tissue infection [164]. Recently, VanderWal and colleagues have shown that PmtA is a Fe(II) exporter and protects GAS against iron intoxication [165]. The expression of *pmtA* was induced by iron and a *pmtA* mutant exhibited increased sensitivity to iron as compared to its parental strain [165].

## 5. TCS and Stand-Alone Regulators as Potential Antibacterial Targets

Prior to an antibacterial drug design, the main question remains: what is an ideal target? The new drug should (i) target essential functions of bacterial viability or pathogenesis; (ii) be specific to minimize side effects on host microbiota; and (iii) be exclusively present in the bacteria of choice in order to reduce potential cytotoxic effects in the host. Bacterial regulators are mostly cytoplasmic molecules, which are responsible for the control of multiple physiological or pathogenic processes. However, experimental evidence clearly shows that targeting just one regulator will not be enough. To achieve bactericidal or bacteriostatic effects comparable to current antibiotic treatment, it will require a multi-targeting approach. However, an alternative approach could also be applied. New therapeutics could target exclusively specific regulators of colonization, pathogenesis, dissemination, or immune evasion in order to disarm a specific characteristic of the pathogen and to enhance its susceptibility to host immune response or to an additive antibiotic treatment [166].

In most cases, the function of transcriptional regulators can be inhibited by small molecules that either sterically block or promote a conformational change of the DNA-binding domain [167] or the dimerization domain [168]. To the best of our knowledge, no such attempts were done in GAS research. However, there are a few successful examples from research with Gram-negative pathogenic bacteria. One example is the master regulator VirF in *Shigella* [167]. Several studies have shown that VirF mutants are avirulent [169,170]. Subsequent screening of 100,000 potential inhibitory small molecule compounds identified only one, namely SE-1 (1-butyl-4-nitromethyl-3-quinolin-2-yl-4*H*-quinoline), which inhibited VirF DNA binding activity, consequently reduced expression of the genes that are under VirF control, and reduced the *S. flexneri* infectivity of mouse fibroblasts [167].

In Gram-positive bacteria, a successful inhibition of the accessory gene regulator (*agr*)-system of *Staphylococcus aureus* was demonstrated. The expression of different virulence factors is regulated by this TCS [67]. Furthermore, it was shown that the entire TCS is a mutational hotspot and these mutations can determine the phenotypic properties of *S. aureus* resulting in invasive or colonizing strains [171]. A study by Sully and colleagues identified savirin (*S. aureus* virulence inhibitor) as a potent inhibitor of AgrA, a two-component sensor regulator [172]. Specifically, savirin inhibited DNA binding activity of AgrA. These actions resulted in reduced transcription of AgrA target genes. Subsequently, several methicillin-resistant and methicillin-sensitive *S. aureus* strains showed reduced hemolytic activity. Moreover, savirin was highly efficacious in two murine skin infection models. Treatment of skin infections with this compound reduced tissue injury and promoted bacterial clearance [172]. Of note, savirin was ineffective against the important skin commensal *Staphylococcus epidermidis* [172]. Comparable approaches are yet to be developed for the fight against *S. pyogenes* infections.

## 6. Conclusions

In summary, GAS express a plethora of virulence factors, which are integrated into a complex regulatory network. These networks have evolved in concert with host niches and challenges the bacteria face during the different stages of infection. The ultimate purpose is to ensure the survival of the pathogen. To fuel the development of new and more effective therapeutics, detailed understanding of GAS regulatory networks of virulence traits and host niche adaptation is warranted. This knowledge may pave the way to develop anti-virulence therapeutics directed against the plethora of virulence regulators of *S. pyogenes* discussed here.

## Data Availability

Not applicable.
